# Supplementary material for: End Sequence Analysis Toolkit (ESAT) expands the extractable information from single-cell RNA-seq data
Source: Genome Res. 2016 Oct;26(10):1397–410. doi: 10.1101/gr.207902.116 (PMC5052061; doi:10.1101/gr.207902.116)
Supplement: Supplemental Material [file supp_gr.207902.116_Supp_Materials.tar.gz › ESAT-esat_v0.1/doc/html/classbroad_1_1core_1_1util_1_1_parse_g_c_t_file-members.html]

ESAT: Member List


ESAT


- Main Page
- Related Pages
- Packages
- Classes
- Files

- Class List
- Class Index
- Class Hierarchy
- Class Members

All Classes Namespaces Files Functions Variables Enumerator Pages

- broad
- core
- util
- ParseGCTFile

broad.core.util.ParseGCTFile Member List

This is the complete list of members for broad.core.util.ParseGCTFile, including all inherited members.

|  |  |  |
| --- | --- | --- |
| getControlClass(File file) | broad.core.util.ParseGCTFile | static |
| getGroupIndexes(ArrayList< String > groups) | broad.core.util.ParseGCTFile | static |
| getGroupIndexes(File clsFile) | broad.core.util.ParseGCTFile | static |
| getIndexGroup(ArrayList< String > groups) | broad.core.util.ParseGCTFile | static |
| getOrderedListCDT(File file) | broad.core.util.ParseGCTFile | static |
| loadData(File file) | broad.core.util.ParseGCTFile | static |
| loadPIDName(File file) | broad.core.util.ParseGCTFile | static |
| logTransform(Map< String, ArrayList< Double >> data, double addFactor) | broad.core.util.ParseGCTFile | static |
| parseCDT(File file) | broad.core.util.ParseGCTFile | static |
| parseChipFile(File file) | broad.core.util.ParseGCTFile | static |
| parseCLS(File file) | broad.core.util.ParseGCTFile | static |
| parseCLSGroups(File file) | broad.core.util.ParseGCTFile | static |
| parseGeneList(File file) | broad.core.util.ParseGCTFile | static |
| parseNumericCls(File file, List< String > header) | broad.core.util.ParseGCTFile | static |
| writeGCT(String save, Map< String, ArrayList > gct) | broad.core.util.ParseGCTFile | static |
| writeGMTFile(String save, Map< String, Collection< String >> gmtFile) | broad.core.util.ParseGCTFile | static |


---

Generated on Fri Aug 15 2014 15:10:57 for ESAT by  

 1.8.7
